# Supplementary material for: Chromothripsis during telomere crisis is independent of NHEJ, and consistent with a replicative origin
Source: Genome Res. 2019 May;29(5):737–49. doi: 10.1101/gr.240705.118 (PMC6499312; doi:10.1101/gr.240705.118)
Supplement: Supplemental Material [file supp_gr.240705.118_Supplemental_file_1.zip › contigs/annotated_contigs/DB46/contig.3.DB46_length_821_mean_cov_8.06090133983.docx]

**DB46_length_821_mean_cov_8.06090133983**

```
GAAGAGTTGAAGTCAAATTATCATTGTTTGCAGATGACATGATCCTATGTCTAGAAAACCACATTGTCACAGCCTGGAACTTCTTAACA
 >chr6:101423753-101424294 + E=1e-307
TGAGAAGCAACTTTCACAAAATTTTAGGATACAAAATGAATGTGTGAAAGTCACTAGCATTCCTATACACCAACAACAGTCAAGCCAAG

AGACAAATCAGGAATGAAATCCCATTCACAATTGTCACAAAAAGAATAGAATACCTAGAAACACAGGTGACAAGGGAAGTGAAGGACCT

TTTCAAGGAGAACTGCAAACCACTGCTCAAATAAATCAGAGAGGGCACAAAGAAATAGAAAAATATTCCATGCTCATAGATAGGAAGAA

TCAATATTGTGAAAATAGCCATACTGCCCAAAGCAGTTTATAGATATATTCCCATTAAACTACCATTGACATTCTTCACAGAATTTGGA

AAAACTATTTTAAAATTCATATGGAACCTGAAAAGAGCCTGAATAGCCAAGACAATCCTAAACGTGAAGAAAGCTGAAGGCATCACACT

ACTCG|AC|ATTCTATTATAAAGATACATGCACACATATGTTCACTGAAGCACTATTCACAATAG|GAAAAATG|TGGAATCAACCTAA
      >chr6:113845403-113845461 - E=5e-05                               >chr8:1298805
ATGCCCATCAATGATAGATTAGATAAAGAAAATGTGGTACATGTACACCATGGAATACTATGCAGCCATAAAAAAGGATGAGTTCATGT
44-129880760 - E=5e-91
CCTTTGCGGGGACAGGGATGAAGCTGGAAACCATCATTCTTAGCAAACACAAGAACAGAAAACCAAACACTGCGTGTTCTCACTCATAA

CTAGGAGTTGAATAATGAGAACAC
```
